# Prevalence of human papillomavirus cervical infection in an Italian asymptomatic population

**DOI:** 10.1186/1471-2334-5-77

**Published:** 2005-09-27

**Authors:** Maria G Centurioni, Andrea Puppo, Domenico F Merlo, Gennaro Pasciucco, Enzo R Cusimano, Rodolfo Sirito, Claudio A Gustavino

**Affiliations:** 1 Department of Surgical Therapies, Istituto Nazionale per la Ricerca sul Cancro, Largo Rosanna Benzi 10, 16100 Genova, Italy; 2 Department of Epidemiology and Prevention, Istituto Nazionale per la Ricerca sul Cancro, Largo Rosanna Benzi 10, 16100 Genova, Italy; 3 Department of Obstetrics and Gynaecology, Ospedale Evangelico Internazionale, Corso Solferino 1, 16100 Genova, Italy

## Abstract

**Background:**

In the last decade many studies have definitely shown that human papillomaviruses (HPVs) are the major cause of cervical carcinogenesis and, in the last few years, HPV testing has been proposed as a new and more powerful tool for cervical cancer screening. This issue is now receiving considerable attention in scientific and non scientific press and HPV testing could be considered the most important change in this field since the introduction of cervical cytology. This paper reports our prevalence data of HPV infection collected in the '90s, while a follow up of these patients is ongoing.

**Methods:**

For this study we used polymerase chain reaction (PCR) to search HPV DNA sequences in cervical cell scrapings obtained from 503 asymptomatic women attending regular cervical cancer screening program in the city of Genova, Italy. All patients were also submitted to a self-administered, standardized, questionnaire regarding their life style and sexual activity. On the basis of the presence of HPV DNA sequences women were separated into two groups: "infected" and "non infected" and a statistical analysis of the factors potentially associated with the infection group membership was carried out.

**Results:**

The infection rate was 15.9% and the most frequent viral type was HPV 16.

**Conclusion:**

Our HPV positivity rate (15.9%) was consistent to that reported by other studies on European populations.

## Background

Although the Pap-test has given a great contribution to early diagnosis of cervical cancer, this is still a very common, worldwide distributed, female neoplasm and a major cause of death in developing countries. Moreover, in developed countries, the treatment of preneoplastic cervical lesions is a considerable public health problem. There is now strong evidence that infection with carcinogenic types of HPV represents a nearly universal event in cervical carcinoma development and sexual transmission is the predominant mode of HPV infection [1-3]. The relative risk of cervical cancer associated with high-risk types of HPV is even higher than the risk of lung cancer associated with smoking. More than 50 HPV types infecting the genital tract have been identified and sequenced and they can be associated with distinct clinical manifestation. Some HPV types, like 6 and 11, are cause of benign condylomas (low risk group) while a wider number of subtypes (HPV 16, 18, 31, 33, 35, 39, 45, 51, 52, 56, 58, 59, 68) has been proved to be involved in cervical carcinogenesis (high risk group). Several oncogenes have been identified in the oncogenic types and the biologic mechanism of malignant transformation has been increasingly well characterized [4].

HPV testing for cancer-associated HPV DNA is now accepted as a viable and validated option in the management of women with equivocal cytologic findings [5-7] and, in the last few years, there has been an increasing interest in using the HPV testing also in cervical samples from asymptomatic women without cytological abnormalities [8-10]. This strategy seems to allow an early identification of populations at different risk level for this neoplasia because the absence of infection makes the risk of cancer negligible. Large-scale screening studies demonstrated that HPV testing is more sensitive for the detection of high-grade cervical lesions than cytology.

Nevertheless, the lower specificity and the high cost of HPV testing make its use in primary cervical cancer screening still questionable. At the same time very promising trials are ongoing to evaluate the efficacy of prophylactic HPV 16 vaccines [11,12]. These results suggest that the increasing knowledge about the importance of HPV infection is probably leading to new prevention strategies for this disease. Polymerase Chain Reaction (PCR) is a sensitive technique for the detection of very small amounts of HPV's nucleic acids in clinical specimens and it has been used in most epidemiological studies that have evaluated the role of these viruses in cervical cancer causation. In the '90s we performed a study of prevalence of this infection in cervical samples obtained from women attending their annual screening. This paper presents our historical data and, in our knowledge, this is the first report regarding an Italian population.

## Methods

**Figure 1:**
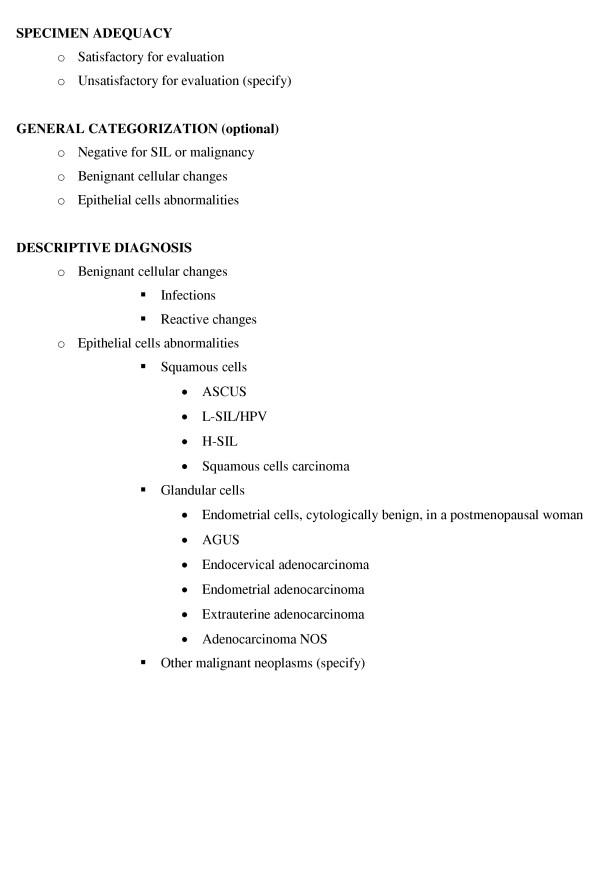
Bethesda System 1991.

This study included 514 women attending the Istituto Nazionale per la Ricerca sul Cancro in Genova (Italy) for their routine annual Pap smear from June 1992 to June 1993. At the time of their visit all the participants were informed of the research and its purpose and gave their informed consent. Then, they were invited to fill in a self administered questionnaire including items about lifetime number of male sexual partners, age at first intercourse, history of pregnancy and sexually transmitted diseases, contraceptives methods, smoking, prior abnormal Pap smears and other variables. Pap smears were all reviewed by the same pathologist according to the Bethesda System 1991 (figure 1) [13], without knowledge of clinical or laboratory data.

### Sample preparation

Cervical specimens were collected with a cotton swab pre-wet with PBS and suspended in a 50 ml conical tube containing 10 ml PBS. The tube was vortexed to remove all the material from the swab, which was discarded. The tube was then centrifuged at 2000 g for 10 min, the supernatant was removed and discarded, the pellet resuspended in 500 μl PBS, transferred into a 1.5 ml Eppendorf tube and frozen at -80°C until DNA extraction was performed with the following procedure: phenol/chlorophorm:isoamyl alcohol 24:1, precipitation with 0.3 M sodium acetate in 2.5 V/V pure ethanol, centrifugation at 14,000 rpm for 20 min, washing with 70% ethanol, resuspension in double distilled water.

### PCR

The consensus primers MY09 and MY11 were used for the amplification in the following 100 μl reaction mixture: 10 μl of 10 × PCR buffer, 0.2 μM of each primer, 200 μM of each dNTP, 2.5 units of Taq polymerase (Perkin Elmer/Cetus), 200 ng of sample DNA, double distilled water. The apmplification was performed in a DNA Thermal Cycler (Perkin Elmer/Cetus Instruments), using the following program set: 95°C for 30 sec., 55°C for 30 sec., 72°C for 1 min. for a total number of 30 cycles plus an additional 9 min. at 72°C. Separate rooms were used for: preparation of DNA template, preparation and storage of reagents, setting up the amplification reaction. All reagents used in PCR were prepared, aliquotted and stored in an area that was free of PCR- amplified products. Amplification of a single copy human gene (β-globin), as control of DNA suitability for amplification, was performed in each reaction tube; we also used He-La cells as positive control and K562 cells as negative control in each reation.

### Analysis of the amplification products

10 μl of the amplification mixture were analysed by electrophoresis in 1.5 agarose gel and visualized by UV light after ethidium bromide staining. Dot-blot analysis was then performed using 2 and 5 μl of the PCR mixture after alkaline denaturation treatment (NaOH 1.5 M and NaCl 0.5 M). DNA was transferred onto nylon membranes Hybond N^+ ^(Amersham, UK) using the Dot-blot apparatus (Millipore, USA) under constant vacuum conditions. Replicate membranes were used for Hybridization with each of the type specific probes (MY12, MY13, MY14, WD74 and MY16) and the generic probe mix (GP1 and GP2). For detection we used the ECL chemoilluminescence kit (Amersham, UK).

### Statistical analysis

The associations between HPV infection (defined as a positive HPV-PCR) and individual covariates, including age, age at first sexual intercourse, marital status, smoking habits, education, number of lifetime partners and use of oral contraceptives was investigated using univariate and multiple logistic regression (MLR) analyses. The analyses were done with the SPSS Statistical Software (SPSS Inc. Chicago Illinois). Odds ratios point estimates (OR) and their 95% confidence intervals (95% CIs) were computed by MLR procedure for binary data to estimate the association between each covariate levels and HPV infections while adjusting for the effect of other variables retained in the model. Statistical tests were considered to be significant if the p value was 0.05 or less.

## Results

Amongst 514 women enrolled in this study, 503 samples were adequate for PCR. The most important patient characteristics are shown in table 1. The mean age was 51 years (SD = 12.3). Our group was heterogeneous for schooling and other socio-economic variables. Three out of 503 women had cytological abnormal findings (SIL) and no one had genital warts on examination at enrollment. As shown in table 2, 80/503 (15.9%) samples were found positive for HPV infection. The distribution of different viral types by age group is shown in table 2. HPV 16 was the most frequently found subtype (46/80, 57.5%). 19/80 (23.7%) samples were positive for more than one type. Patients carrying a SIL (3/503) were all found positive for HPV (2 for type 16; 1 for type 18+33). The results of the univariate and multiple logistic regression analyses are shown in table 3 and 4, respectively. The number of male lifetime partners was the only covariate significantly associated with HPV infection in univariate analysis (p = 0.03). In multiple regression analysis increased ORs were found to be associated with higher levels of education (p trend = 0.03) while none of the other covariates, including the number of male lifetime partners, was associated with HPV infection.

**Table 1:** Distribution of selected characteristics observed in the 503 patients included in the study.

**N°**		503	
**Age**	**Mean (s.d.)**	51 (12.3)	
	**Range**	20–81	
**Age at first intercourse**	**Mean (s.d.)**	20.8 (6.3)	
	**Range**	5*-38	
**Marital status**	**Single**	123	(24.45%)
	**Married**	369	(73.36%)
	**Unknown**	11	(2.19%)
**Education**	**High school or University**	207	(41.1%)
	**Middle School**	167	(33.2%)
	**Primary School**	120	(23.8%)
	**Unknown**	9	(1.9%)
**History of pregnancy**	**0**	90	(17.8%)
	**1–2**	263	(52.2%)
	**≥ 3**	137	(27.2%)
	**n.a.**	13	(2.5%)
**N° of lifetime partners**	**1**	294	(58.45%)
	**2–4**	74	(14.71%)
	**5–9**	33	(6.56%)
	**≥ 10**	47	(9.34%)
	**Unknown**	55	(10.93%)
**History of sexually transmitted diseases**	**No**	479	(95.2%)
	**Yes**	24	(4.8%)
**Smoking**	**No**	348	(69.2%)
	**Yes**	155	(30.8%)
**Oral contraceptives**	**No**	337	(67%)
	**Yes/ex**	166	(33%)
**History of abnormal Pap-smear**		50/503	(9.94%)
**SIL in the present Pap smear**		3/503	(0.6%)

* sexual abuse

**Table 2:** Viral type distribution by age group assessed by PCR and dot-blot analyses. The percentage of PCR and dot-blot positive samples (among HPV-PCR +) are shown in parenthesis.

	**PCR analysis**	**Dot-blot analysis**					
**Age**	**HPV (all)**	**Generic probe**	**6/11**	**16**	**18**	**33**	**Others**

≤ 44	18/119 (15.1%)	16 (88.9%)	1 (5.6%)	8 (44.4%)	4 (22.2%)	2 (11.1%)	2 (11.1%)
45–52	21/120 (17.5%)	19 (90.5%)	2 (9.5%)	10 (47.6%)	11 (52.4%)	3 (14.3%)	2 (9.5%)
53–59	14/122 (11.5%)	13 (92.9%)	0	11 (78.6%)	4 (28.6%)	0	1 (7.1%)
≤ 60	27/142 (19.0%)	26 (96.3%)	1 (3.7%)	17 (63.0%)	10 (37.0%)	2 (7.4%)	2 (7.4%)
**All**	**80/503 (15.9%)**	**74 (92.5%)**	**4 (5.0%)**	**46 (57.5)**	**29 (36.3)**	**7 (8.8%)**	**7 (8.8%)**

**Table 3:** Findings from the univariate analysis: HPV positive patients by selected covariates

**Covariates**		**All Women**	**HPV Positive**		**P value χ^2 ^test**
		**No.**	**No.**	**%**	

**Age**	≤ 44	119	18	15.1	0.37
	45–52	120	21	17.5	
	53–59	122	14	11.5	
	≥ 60	142	27	19.0	
**Education**	Primary School	120	12	10.0	0.09
	Secondary School	167	27	16.2	
	Higher	207	40	19.3	
	Unknown	9	-	-	
**Smoking habits**	Current + previous	155	26	16.8	0.79
	Never smokers	348	54	15.5	
**N° of lifetime partners**	1	293	35	11.9	0.03
	> 1 ≤ 5	74	13	17.6	
	> 5	78	19	24.4	
	Unknown	58	12	20.0	
**Age at 1° sexual intercourse**	≤ 18	128	22	17.2	0.63
	19 – 21	138	24	17.4	
	22 – 24	109	13	11.9	
	≥ 24	125	19	15.2	
**Marital status**	Single	123	27	21.95	0.06
	Married	369	52	14.1	
	Unknown	11	-	-	
**Use of Oral Contraceptives**	Current + previous	166	27	16.3	0.88
	Never	337	53	15.7	

**Table 4:** Findings from multiple logistic regression: Odds Ratios (OR) point estimates and their 95% Confidence Intervals (95% C.I.)

**Covariate**	**No.**	**OR ^(a)^**	**95% C.I.**	**P ^(b)^**	**P trend^(c)^**
**Age**					
≤ 44	106	1 (ref.)		0.21	0.13
45 – 52	113	1.88	0.80 – 4.41		
53 – 59	103	1.20	0.45 – 3.18		
≥ 60	110	2.29	0.89 – 5.89		
**No. of lifetime partners**					
1	283	1 (ref.)		0.29	0.11
1 – 5	71	1.30	0.61 – 2.78		
> 5	78	1.82	0.86 – 3.85		
**Use of Oral Contraceptives.**					
No	284	1 (ref.)		0.64	
Yes	148	1.16	0.61 – 2.21		
**Education**					
Primary	93	1 (ref.)		0.44	0.03
Secondary	153	1.39	0.61 – 3.16		
Higher	186	1.72	0.73 – 4.03		
**Smoking**					
No	290	1 (ref.)		0.92	
Yes	142	0.97	0.54 – 1.74		
**Age 1° intercourse**					
**≤ 18**	94	1 (ref.)		0.74	0.63
**19 – 21**	124	1.04	0.48 – 2.24		
**22 – 24**	100	0.70	0.28 – 1.70		
**> 24**	114	0.77	0.32 – 1.86		
**Marital status**					
Single	101	1 (ref.)		0.31	
Married	331	0.71	0.37 – 1.36		

The analysis is based on 432 women with complete data; all variables included in the model. ^(a) ^(ref.) = reference level; ^(b) ^Wald test for the contribution of the covariate to the logistic model; ^(c) ^Test for linear trend within individual categorical covariates.

## Discussion

In the nineteenth century some investigators noticed that cervical cancer was almost exclusively detected in married women, being rare in the unmarried ones and exceptional in nuns [20,21]. Since then, a large number of studies have investigated the importance of sexual activity as the main risk factor for cervical carcinoma development (increased risk related to early onset and number of lifetime partners), suggesting a role for a sexually transmitted infectious agent. More recently, many epidemiological studies have identified HPV as the main factor in cervical cancer causation [14,22]. At the time of this study we wanted to investigate whether there was any association between genital HPV infection and known risk factors for cervical cancer. The sample we studied was a non selected population attending our hospital from the whole Liguria Region, in Italy. Our HPV positivity rate (15.9%) was low compared to that detected by the first studies conducted using PCR [14,23], but it matches well with prevalence data more recently detected in other European countries [8,15,24,25]. A significant relationship was observed in our population between age and HPV infection. Moreover, in the group aged ≤ 30 (33 women) the HPV prevalence was higher (27.3% vs 15.1%, data not shown) and similar to that reported by other investigators [14-19]. The different HPV frequency between age groups could be due to their different sexual behaviour, although this cannot completely explain the findings of the intermediate age group (>30 y.o., <50 y.o.). In fact the sexual habits of this population, as confirmed by the questionnaires, are similar to those of younger subjects (<30 y.o.), but HPV is detectable in a lower rate (16%), close to the value found for the older group. A better explanation of these results, also reported by other studies [18,26], is that most HPV genital infections are transient because they are cleared by host immune response. In fact serum IgG antibodies develop in response to infection in 55–92% of women [12,27]. These data are very helpful now that the introduction of HPV testing in cervical cancer screening seems to be incoming, and new guidelines are needed. Our data support the evidence that this kind of screening should not start before age 30 (later in comparison to the cytology – based screening), because in young women HPV infection is too frequent [8,19,24].

In our population the "higher education" was the most represented group (205/436 subjects), and this may have affected the observed association with HPV infection, due to a an easier access to health system services.

According to other studies, the most frequent viral type was HPV 16 [15], an important finding when planning new prevention strategies (e.g. prophylactic vaccines). Smoking and use of oral contraceptives, previously reported to be risk factors for cervical carcinoma development, were not related to HPV infection in our data. A major confounding factor of the present investigation is the lack of objective and anamnestic data regarding male partners. Moreover other factors (such as the immune response to the HPV) still need to be better evaluated.

## Competing interests

The author(s) declare that they have no competing interests.

## Authors' contributions

MGC conceived of the study, carried out part of the molecular analysis and drafted the manuscript, AP helped to draft the manuscript, DFM performed the statistical analysis, GP carried out most of the molecular assays, ERC and RS enrolled the patients, CAG participated in the design and coordination of the study and helped to draft the manuscript.

## Pre-publication history

The pre-publication history for this paper can be accessed here:



## References

[B1] Mc Cance DJ, Campion MJ, Clakson PK, Chesters PM, Jenkins D, Singer A (1985). Prevalence of human papillomavirus type 16 DNA in cervical intraepithelial neoplasia and invasive carcinoma of the cervix. Br J Obstet Gynaecol.

[B2] Franco E, Bergeron J, Villa L, Arella M, Richardson L, Arseneau J, Stanimir G (1996). Human Papillomavirus DNA in Invasive Cervical Carcinomas and Its Association with Patient Survival: a Nested Case-Control Study. Cancer Epidemiol Biomarkers Prev.

[B3] Wolf JK, Franco EL, Arbeit JM, Shroyer KR, Wu TC, Runowicz CD, Tortolero Luna G, Herrero R, Crum CP (2003). Innovation in Understanding the Biology of Cervical Cancer. Cancer Suppl.

[B4] Matlashewski G, Schneider J, Banks L, Jones N, Murray A, Crawford L (1987). Human papillomavirus type 16 DNA co-operates with activated *ras *in transforming primary cells. EMBO J.

[B5] Arbyn M, Buntinx F, Van Ranst M, Paraskevaidis E, Martin-Hirsch P, Dillner J (2004). Virologic Versus Cytologic Triage of Women Eith Equivocal Pap Smears; a Meta-analysis of the Accuracy To Detect High-Grade Intraepithelial Neoplasia. J Natl Cancer Inst.

[B6] Arnold K (2001). Study Results Help Define HPV's Role as Diagnostic Tool. J Natl Cancer Inst.

[B7] Solomon D, Schiffman M, Tarone R (2001). Comparison of Three Management Strategies for Patients With Atypical Squamous Cells of Undetermined Significance: Baseline Results From a Randomized Trial. J Natl Cancer Inst.

[B8] Petry KU, Menton S, Menton M, van Loenen-Frosch F, de Carvalho Gomes H, Holz B, Schopp B, Garbrecht-Buettner S, Davies P, Boehmer G, van den Akker E, Iftner T (2003). Inclusion of HPV testing in routine cervical cancer screening for women above 29 years in Germany: results for 8466 patients. Br J Cancer.

[B9] Wright TC, Schiffman M (2003). Adding a Test for Human Papillomavirus DNA to Cervical-Cancer Screening. N Engl J Med.

[B10] Cuzick J, Szarewski A, Cubie H, Hulman G, Kitchener H, Luesley D, McGoogan E, Menon U, Terry G, Edwards R, Brooks C, Desai M, Gie C, Ho L, Jacobs I, Pickles C, Sasieni P (2003). Management of women who test positive for high-risk types of human papillomavirus:the HART study. Lancet.

[B11] Koutsky LA, Ault KA, Wheeler CM, Brown DR, Barr E, Alvarez FB, Chiacchierini LM, Jansen KU (2002). A controlled trial of a human papillomavirus type 16 vaccine. N Engl J Med.

[B12] Galloway DA (2003). Papillomavirus vaccines in clinical trials. Lancet Infectious Diseases.

[B13] (1992). The Bethesda System for Reporting Cervical/Vaginal Cytologic Diagnoses (1992) Report of the 1991 Bethesda Workshop. Am J Surg Pathol.

[B14] Ley C, Bauer HM, Reingold A, Schiffman MH, Chambers JC, Tashiro CJ, Manos MM (1991). Determinants of genital human papillomavirus infection in young women. J Natl Cancer Inst.

[B15] Jacobs MW, Walboomers JM, Snijders PJ, Voorhorst FJ, Verheijen RH, Fransen-Daalmeijer N, Meijer JL (2000). Distribution of 37 mucosotropic HPV types in women with cytologically normal cervical smears: the age-related patterns for high-risk and low-risk types. Int J Cancer.

[B16] Cuschieri KS, Cubie HA, Whitley MW, Seagar AL, Arends MJ, Moore C, Gilkisson G, McGoogan E (2004). Multiple high risk HPV infections are common in cervical neoplasia and young women in a cervical screening population. J Clin Pathol.

[B17] Dalstein V, Riethmuller D, Prétet J-L, Le Bail Carval K, Sautière J-L, Carbillet J-P, Kantelip B, Schaal JP, Mougin C (2003). Persistence and load of high-risk HPV are predictors for development of high-grade cervical lesions: a longitudinal french cohort study. Int J Cancer.

[B18] Evander M, Edlund K, Gustafsson A, Jonsson M, Karlsson R, Rylander E, Wadell G (1995). Human papillomavirus infection is transient in young women: a population based cohort study. J Infect Dis.

[B19] Peto J, Gilham C, Deacon J, Taylor C, Evans C, Binns W, Haywood M, Elanko N, Coleman D, Yule R, Desai M (2004). Cervical HPV infection and neoplasia in a large population-based prospective study: the Manchester cohort. Br J Cancer.

[B20] Rigoni Stern D (1842). Fatti statistici relativi alle malattie cancerose. Giorn, Prog Patol Terap.

[B21] Skrabanek P (1988). Cervical cancer in nuns and prostitutes: a plea for scientific continence. J Clin Epidemiol.

[B22] Daling JR, Madeleine MM, McKnight B, Carter JJ, Wipf GC, Ashley R, Schwartz SM, Beckmann AM, Hagensee ME, Mandelson MT, Galloway DA (1996). The Relationship of Human Papillomavirus-related Cervical Tumors to Cigarette Smoking, Oral Contraceptive Use, and Prior Herpes Simplex Virus Type 2 Infection. Cancer Epidemiol Biomarkers Prev.

[B23] Bauer HM, Ting Y, Greer CE (1991). Genital human papillomavirus infection in female university students as determined by a PCR-based method. JAMA.

[B24] Baay MF, Tjalma WA, Weyler J, Goovaerts G, Buytaert P, Van Marck EA, Lardon F, Vermorken JB (2001). Human papillomavirus infection in the female population of Antwerp, Belgium: prevalence in healthy women, women with premalignant lesions and cervical cancer. Eur J Gynaecol Oncol.

[B25] Forslund O, Antonnson A, Edlund K, van den Brule AJ, Hansson B-G, Meijer CJ, Ryd W, Rylander E, Strand A, Wadell G, Dillner J, Johansson B (2002). Population-Based Type-Specific Prevalence of High-Risk Human Papillomavirus Infection in Middle-Aged Swedish Women. J Med Virol.

[B26] Kang m, Lagakos SW (2004). Evaluating the role of human papillomavirus vaccine in cervical cancer prevention. Stat Methods Med Res.

[B27] Wikstrom A, van Doornum GJ, Quint WG, Schiller JT, Dillner J (1995). Identification of human papillomavirus seroconversions. J Gen Virol.

